# Interindividual Variability in Thyroid Cartilage Lamina Width and Its Implications for Personalized Medialization Thyroplasty

**DOI:** 10.3390/jpm16060294

**Published:** 2026-05-29

**Authors:** Mar Gimeno-Coret, Natsuki Oishi, Rosa Hernández-Sandemetrio, Enrique Zapater

**Affiliations:** 1 ENT Department, General University Hospital of Valencia, 46014 Valencia, Spain; gimeno_marcor@gva.es; 2 Faculty of Medicine and Odontology, Valencia University, 46010 Valencia, Spain; rosa.hernandez@uv.es (R.H.-S.); enrique.zapater@uv.es (E.Z.)

**Keywords:** thyroid cartilage, gender, anatomical variability, laryngeal framework surgery

## Abstract

**Aim**: The aim of this study was to analyze anterior and posterior thickness measurements of the thyroid cartilage lamina and to assess their association with age and sex, with a focus on interindividual anatomical variability relevant to personalized medialization thyroplasty. **Methods**: A retrospective observational study was conducted on 47 patients with unilateral vocal cord paralysis who were candidates for medialization thyroplasty. The thickness of the thyroid cartilage was measured at its anterior and posterior aspects on an axial CT scan at the level of the glottic plane. The Wilcoxon signed-rank test was used to compare anterior and posterior thickness measurements, and a generalized linear mixed model (was fitted to assess the influence of anatomical region, age, and sex on cartilage thickness, with patient as a random effect. **Results**: The posterior thickness was significantly greater than the anterior thickness (median difference = 1 mm; *p* < 0.001). No significant differences were found in the magnitude of this difference by gender (*p* = 0.37) or a significant correlation with age (r = 0.16; *p* = 0.28). The mixed model confirmed that anatomical region was the only statistically significant fixed effect: the posterior region showed a Risk Ratio of 1.71 (95% CI: 1.26–2.32; *p* < 0.001) relative to the anterior region, indicating that the posterior thickness was approximately 71% greater than that of the anterior region. The interaction between cartilage thickness, age and gender was not statistically relevant. **Discussion**: In our sample, the thyroid cartilage had a thicker posterior width in both sexes. These findings underscore the importance of individualized radiological assessment in laryngeal framework surgery and support a personalized approach to implant selection and surgical planning in medialization thyroplasty.

## 1. Introduction

Vocal fold medialization surgery is a well-established procedure aimed at restoring essential laryngeal functions, primarily voice production and airway protection during swallowing. It is most commonly recommended in patients with unilateral vocal fold paralysis, a condition in which one vocal fold fails to move toward the midline, resulting in incomplete glottic closure and functional impairment. Patients may experience dysphonia, ineffective cough, and aspiration-related swallowing difficulties.

**Figure 1:**
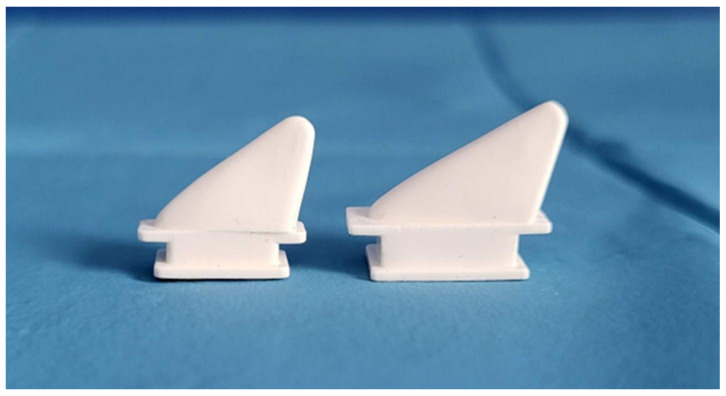
Montgomery prostheses (Boston Medical Products^®^ Shrewsbury, MA, USA) for women (**left**) and men (**right**), of the same size.

One of the most used techniques to solve the symptoms of vocal cord paralysis is the Montgomery medialization thyroplasty [1]. In this procedure, a window is created in the thyroid cartilage to allow the placement of a preformed silicone prosthesis whose size is chosen according to gender, in order to medialize the paralyzed vocal cord. This prosthesis is made up of two main portions: the endolaryngeal portion, which will approach the focal fold to the midline; and the base, which will engage with the thyroid lamina for stabilization of the implant (Figure 1).

The thyroid cartilage is a key anatomical structure in the larynx. Its framework is made of two lamina that have fused anteriorly to form the superior thyroid notch, as well as two superior and inferior cornu, with the latter forming the cricothyroid joint. Its morphology and angulation can vary based on factors such as age, gender and other variables [2,3,4,5,6].

However, increasing amounts of evidence suggests that the morphology of the thyroid cartilage demonstrates substantial interindividual variability that may not be fully explained by sex alone. Such variability may have direct implications for implant positioning, stability, and overall surgical outcomes in laryngeal framework surgery [7,8,9].

While previous anatomical and imaging studies of the laryngeal skeleton have largely focused on sex-based differences [2,3,4,6,10], such an approach provides only a partial understanding of the anatomical factors relevant to surgical decision-making. From a personalized medicine perspective, our clinical experience suggests that the laryngeal skeletal framework demonstrates substantial individual variability that extends beyond gender-related distinctions [9,11].

In this context, radiological assessment using computed tomography (CT) provides a valuable tool for evaluating patient-specific laryngeal anatomy and may support a more individualized surgical approach. Our hypothesis is based on the variability of the thyroid lamina thickness, which may cause incorrect positioning of Montgomery prosthesis, leading to its extrusion or intrusion in the glottic plane. These patient-specific differences in thyroid cartilage morphology and geometry may have a direct impact on surgical strategy and functional outcomes in medialization thyroplasty.

Accordingly, this study aims to provide radiological data that support an individualized anatomical assessment, contributing to precision-oriented planning and optimization of laryngeal framework surgery. Specifically, the aim of this study was to analyze the thickness measurements in the anterior and posterior aspects of the thyroid cartilage and to explore their association with age and gender.

## 2. Methods

**Surgical Context and Rationale:** In Montgomery’s thyroplasty, a window is created in the thyroid plate, where the prosthesis will be placed. This window is located using the inferior thyroid tubercule, a prominent landmark located on the inferior base of the thyroid cartilage. When the location is determined, the window is drilled on the thyroid cartilage. Posteriorly, a premade prosthesis is set in place; said prosthesis is chosen after some intraoperative voice tests using measuring devices that simulate the midline approach of the vocal fold [1].

**Figure 2:**
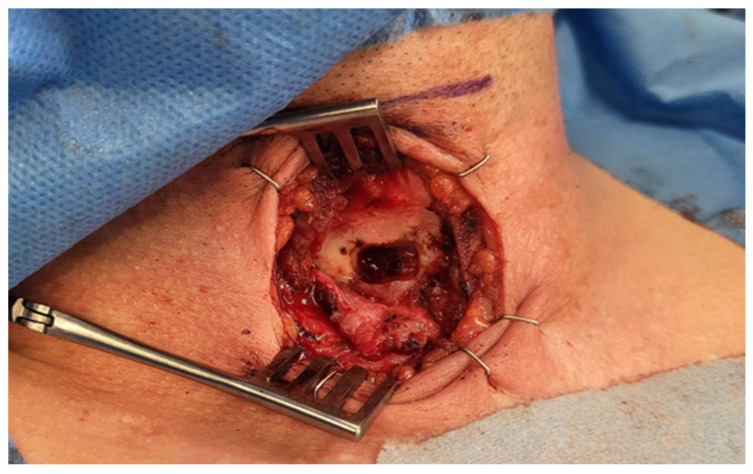
Fenestrated window in the thyroid cartilage where the Montgomery prosthesis is inserted.

The functional outcome of this surgery is observed as an improvement in the voice and fatigue when speaking, and an improvement in swallowing. Observing some suboptimal results in previous classic Montgomery thyroplasties, the last author has attempted to improve these outcomes by modifying the technique to personalize the placement of laryngeal prostheses [7,9], with excellent results. The window was reallocated to a more inferior and anterior location, which was perceived as more suitable for the patients, with a personalized approach [9] (Figure 2).

Thirteen years ago, we described a surgical procedure that used a set of measurement devices designed to achieve accurate individualized placement of the Montgomery prosthesis [7,9]. A personalized surgical approach is essential to optimize vocal and swallowing outcomes, underscoring the relevance of individualized anatomical assessment in medialization laryngeal framework surgery.

**Figure 3:**
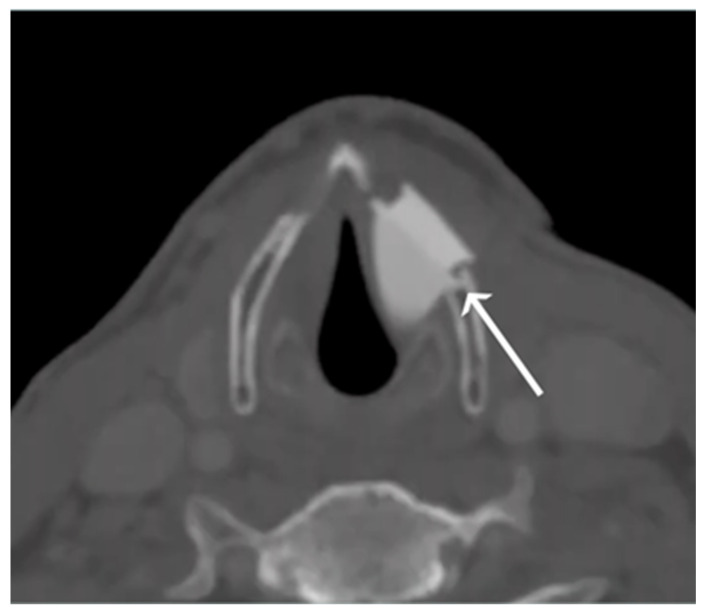
CT imaging of malpositioning of laryngeal prostheses: White arrow showing lack of proper engagement with the thyroid lamina in the posterior area.

**Figure 4:**
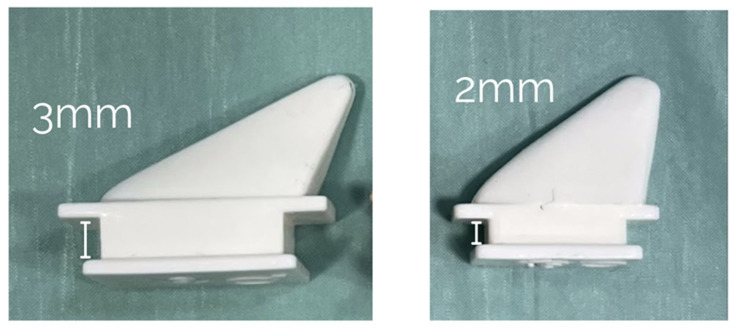
Montgomery prosthesis (Boston Medical Products^®^): on the (**left**), male prosthesis (size 9); on the (**right**), female prosthesis (size 9). The distance between both wings (interala space) on the base is 3 mm in the male prosthesis and 2 mm in the female prosthesis.

During long-term follow-up of these patients, we have perceived prostheses malpositioning in some cases (Figure 3) and have sought to analyze why. One possibility would be that the thickness of the thyroid lamina is not constant; thus, an anatomical variation between the anterior and posterior thickness of the thyroid lamina would be relevant to the results. This was not considered when designing the Montgomery prostheses, which have a constant width on the interala space (Figure 4).

**Study Design and Population:** A retrospective observational study was conducted using CT images of 47 patients aged 17 to 82 years with unilateral vocal cord paralysis who were candidates for medialization thyroplasty. The sample included 23 female and 24 male patients.

**Imaging Acquisition and Measurement Protocol:** All CT scans were obtained as part of routine clinical evaluation using multidetector CT systems (General Electric^®^, Boston, MA, USA). Imaging parameters were standardized as follows: slice thickness: 0.6–1.25 mm; reconstruction: high-resolution bone algorithm; imaging plane: axial acquisition with multiplanar reconstruction and window settings: bone window. Only scans with sufficient image quality and without significant motion or metallic artifacts affecting the laryngeal framework were included.

**Figure 5:**
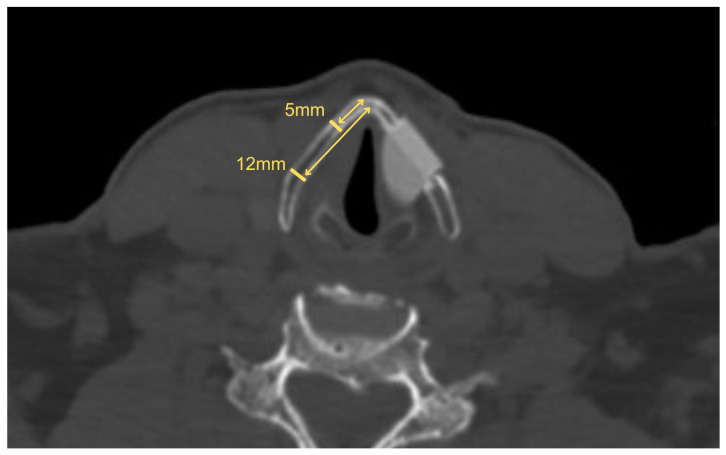
Measurements taken on the anterior and posterior aspects of the thyroid lamina. Anterior: 5 mm from the anterior commissure. Posterior: 12 mm from the anterior commissure.

Thyroid lamina thickness was measured at its anterior and posterior positions on the axial CT scan at the level of the glottic plane—5 mm and 12 mm from the anterior commissure—matching Montgomery’s theoretical window (Figure 5). All measurements were obtained using calibrated digital measurement tools within the radiological workstation. Care was taken to ensure that measurements were performed perpendicular to the external cortical surface of the thyroid lamina to minimize angular bias.

**Measurement strategy:** In 7 patients with preoperative CT scans, bilateral measurements were performed. In the remaining 40 patients, CT imaging was obtained after medialization thyroplasty; therefore, measurements were limited to the contralateral (non-operated) side to avoid distortion related to surgical modification of the cartilage.

This combined approach was adopted to maximize sample size while preserving measurement reliability. However, potential bias related to asymmetry between hemilarynges is acknowledged and addressed in the limitations section.

**Variables:** The primary outcome variable was thyroid cartilage lamina thickness (mm), measured at anterior and posterior locations. A derived variable, defined as the difference between posterior and anterior thickness, was calculated to assess regional variability. Independent variables included age (continuous) and sex (categorical).

Prior to analysis, normality of the distribution was evaluated using the Shapiro–Wilk test for the anterior thickness (W = 0.820, *p* < 0.001), posterior thickness (W = 0.808, *p* < 0.001), and their difference (W = 0.724, *p* < 0.001), confirming a significant departure from normality in all three variables. Accordingly, non-parametric methods were applied throughout. Differences between anterior and posterior thickness were assessed using the Wilcoxon signed-rank test, with results reported as medians and interquartile ranges (IQR).

**Statistical Analysis:** Statistical analyses were performed using R statistical software (version 4.4.3; R Core Team, 2025; R Foundation for Statistical Computing, Vienna, Austria) [10] and RStudio (version 2024.12.1+563; Posit Software, PBC, Boston, MA, USA) [11].

The Wilcoxon signed-rank test was applied to compare anterior and posterior thickness measurements within subjects, given the confirmed non-normal distribution of the data. The association between the thickness difference and sex was assessed using the Mann–Whitney U test, and the correlation with age was evaluated using Spearman’s rank correlation.

To evaluate the simultaneous influence of anatomical region (anterior vs. posterior), age, and sex on cartilage thickness, a generalized linear mixed model (GLMM) was fitted using a Poisson family with log link, given the count-like and non-normally distributed nature of the outcome variable. Patient identifier was included as a random effect to account for the paired structure of the data. Model comparison was performed using likelihood ratio tests (LRTs). Regression coefficients were exponentiated and reported as Risk Ratios (RRs) with 95% Wald confidence intervals.

A post hoc power analysis was conducted based on the primary outcome, showing statistical power >80%. Model assumptions were assessed using standard diagnostic plots. Statistical significance was set at *p* < 0.05. A more detailed description of the statistical models and full outputs are provided in the Supplementary Material.

## 3. Results

**Figure 6:**
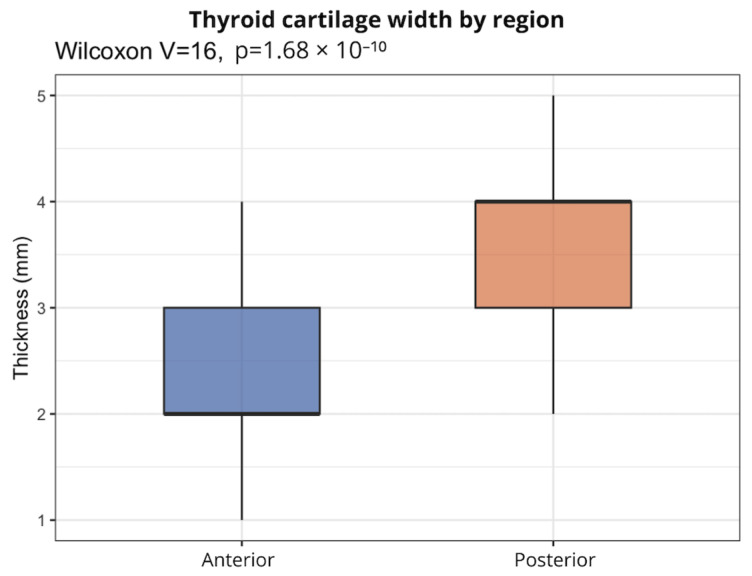
Boxplot diagram where we can observe the difference between anterior and posterior thyroid cartilage width. The posterior aspect of the thyroid lamina is wider than the anterior aspect. Anterior—blue box. Posterior—orange box.

In our sample, the posterior width of the thyroid cartilage was consistently greater than the anterior width. Given the non-normal distribution of the data (Shapiro–Wilk test: W = 0.724, *p* < 0.001 for the difference), non-parametric analyses were performed. The median anterior width was 2.0 mm (IQR: 2.0–3.0) and the median posterior width was 4.0 mm (IQR: 3.0–4.0), yielding a median difference of 1.0 mm (IQR: 1.0–2.0). This difference was statistically significant (Wilcoxon signed-rank test: W = 16.0, *p* < 0.001) (Figure 6).

In order to study the association of the thyroid cartilage width and other variables, we considered the difference in the anterior and posterior aspects of the thyroid cartilage width to obtain a width difference. This variable was studied with regard to gender and age.

**Figure 7:**
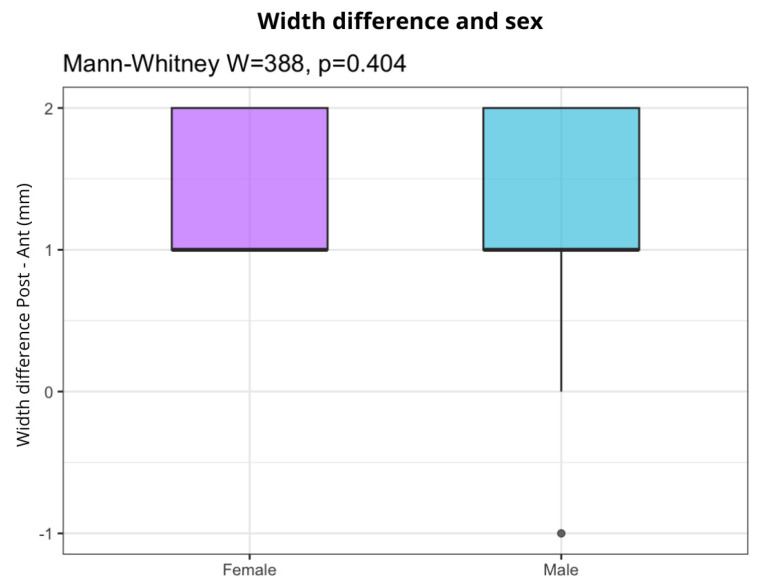
Boxplot graph where we can see no differences between sex and width difference. Female—purple box. Male—blue box.

**Figure 8:**
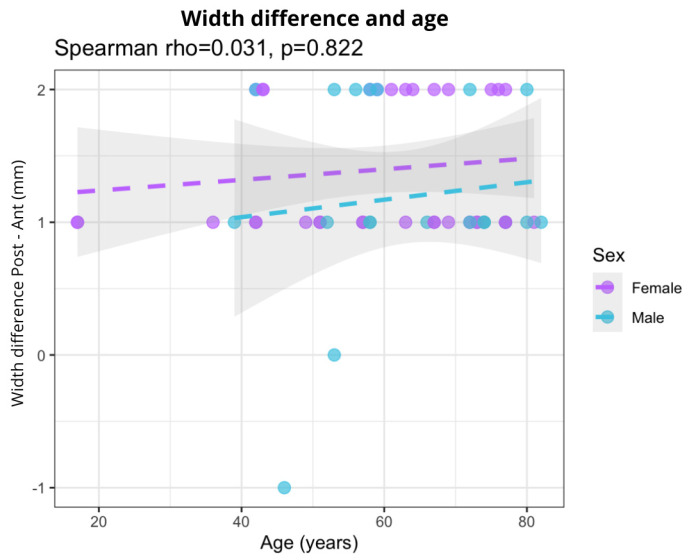
Linearity graph where we can see the lack of linearity between the variables age and thyroid cartilage width difference.

The association between the width difference and sex was assessed using the Mann–Whitney U test. No statistically significant difference was found between female (median = 1.0 mm, IQR: 1.0–2.0) and male patients (median = 1.0 mm, IQR: 1.0–2.0) (U = 383.5, *p* = 0.463) (Figure 7). Similarly, Spearman’s rank correlation revealed no significant association between age and the width difference (rho = 0.071, *p* = 0.608) (Figure 8).

**Figure 9:**
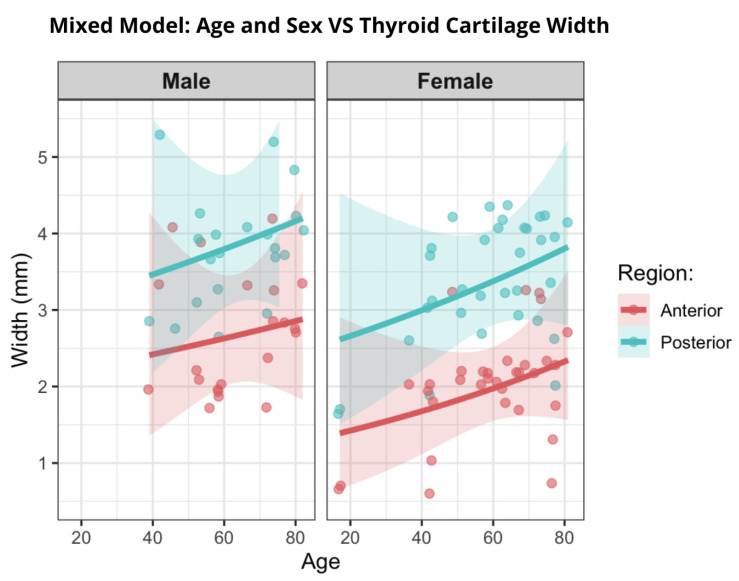
Mixed model showcasing the association between gender, age, thyroid cartilage width and region.

To simultaneously evaluate the effect of anatomical region, age, and sex on cartilage thickness while accounting for the paired structure of the data, a generalized linear mixed model (GLMM) with Poisson family and log link was fitted, with patient as a random effect (Figure 9).

The model confirmed that the anatomical region was the only statistically significant fixed effect: the posterior region showed a Risk Ratio of 1.71 (95% CI: 1.26–2.32; *p* < 0.001) relative to the anterior region, indicating that posterior thickness was approximately 71% greater than the anterior region. Neither age (RR = 1.007; 95% CI: 0.994–1.019; *p* = 0.296) nor sex (RR = 1.317; 95% CI: 0.918–1.889; *p* = 0.135) reached statistical significance, nor did their interactions with region (region × age: *p* = 0.885; region × sex: *p* = 0.494). Likelihood ratio testing confirmed that adding interaction terms did not significantly improve model fit over a model with main effects only (χ^2^(2) = 0.526, *p* = 0.769).

The posterior width was significantly greater than the anterior width in both sexes (*p* < 0.001). While the model estimate for sex suggested a numerically higher overall thickness in male patients (RR = 1.317), this difference did not reach statistical significance (*p* = 0.135), and no significant interaction between sex and anatomical region was identified (*p* = 0.494). Age showed no significant effect on cartilage thickness in either region (*p* = 0.296), and no significant age-by-region interaction was observed (*p* = 0.885).

A post hoc power analysis was performed based on the primary outcome (difference between anterior and posterior thyroid cartilage thickness). Considering the observed mean difference, the study demonstrated adequate statistical power (>80%) to detect the reported effect size at a significance level of 0.05. Nevertheless, given the relatively small sample size, caution is warranted when extrapolating these findings to broader populations.

## 4. Discussion

The present study demonstrates a consistent and statistically significant difference between anterior and posterior thyroid cartilage lamina thickness, with the posterior region being thicker across both sexes. This finding highlights a previously underexplored aspect of laryngeal framework anatomy that may have direct implications for medialization thyroplasty.

Laryngeal framework variability has been widely discussed, including sex-related anatomical differences and their implications for surgery [2,4,6]. These sex-related disparities in the absolute dimensions of the thyroid cartilage are primarily attributed to pubertal anteroposterior growth of the male larynx, as well as differences in the angulation of the thyroid cartilage alae.

In our group, we have focused on anatomical interindividual variability and its potential impact in laryngeal framework surgery. We concluded that it was significant, and its differences are more evident in female specimens [9].

Our findings are consistent with other projects such as the work of Sprinzl et al., who showed a clear asymmetry of the laryngeal framework that evidently affected possible surgical outcomes [4]. In their investigation on the anatomy of the human larynx, they also illustrated the width of the thyroid lamina in the glottic plane; however, they did not highlight its surgical implications.

Vadgaonkar et al. published a morphometrical study of the laryngeal cartilages that also highlighted the variability between specimens; however, in the analysis of the width of the thyroid lamina, there is no specification between the anterior and posterior width [2]. Sahin et al. [6] studied the radiological aspects of laryngeal framework anatomy, also showing the diversity in the patients observed. Bilgen et al. presented a morphometrical study of the laryngeal cartilages, with emphasis on specimen irregularities, especially between male and female subjects, but did not find any correlations with age; this was also supported by our data [3]. Jotz et al. illustrated disparities between male and female larynxes [12], as well as Desuter, specifically targeting diverse outcomes after Montgomery thyroplasty in men and women, attributing these results to anatomical differences [13].

Computed tomography (CT) is a well-established and widely used imaging modality for evaluating the laryngeal framework. Its high spatial resolution and ability to provide detailed visualization of cartilaginous and ossified structures make it particularly suitable for morphometric analysis of the laryngeal skeleton. Furthermore, it plays a key role in preoperative planning by enabling the evaluation of patient-specific structural variations, which are especially relevant in the context of laryngeal framework surgery and personalized approaches to medialization thyroplasty [14,15,16].

From a clinical perspective, analysis of our sample revealed a greater thickness of the thyroid lamina at the posterior aspect compared with the anterior aspect. These differences can result in an inadequate insertion of the prosthesis in the thyroid lamina, as the prosthesis does not take these differences into account (Figure 4). To our knowledge, we have not found any study in the literature that highlights the lack of constant width within the thyroid lamina.

From a clinical perspective, the observed difference between anterior and posterior thyroid cartilage thickness may have implications for implant positioning and stability during medialization thyroplasty. A relatively thinner anterior lamina combined with a thicker posterior region could contribute to suboptimal seating of prefabricated implants. This mismatch may potentially lead to inadequate posterior engagement or altered implant orientation, which could affect surgical outcomes in selected cases.

In line with previous findings on laryngeal framework studies, we have observed interindividual and intergender variability. Based on this conclusion, perhaps the use of prefabricated prosthesis based on gender may not be the best option. We have published our experience personalizing the location of the prosthesis window based on individual laryngeal anatomy, without taking into account the patient’s gender, reinforcing the idea that the surgical treatment of patients with vocal cord paralysis should be personalized, regardless of gender [9]. Based on previous studies where male patients who underwent type I thyroplasty showed major improvement over female patients [7,9], we have recently employed and presented the use of male Montgomery prosthesis in females during revision surgeries in order to achieve similar results as those achieved for male patients [17], highlighting the importance of surgery personalization.

This study has several limitations that should be considered when interpreting the results. First, its retrospective design may introduce selection bias and limits the ability to establish causal relationships. Second, the sample size is relatively small, which may reduce statistical power, particularly for subgroup analyses. Third, the study combines bilateral and unilateral measurements. Although this approach allowed the inclusion of a larger number of patients, it assumes a degree of symmetry between hemilarynges that may not be present in all individuals, potentially introducing bias. Fourth, measurements were obtained exclusively from CT imaging, without direct intraoperative validation. While CT provides high-resolution anatomical detail, minor discrepancies between radiological and in vivo measurements cannot be excluded. Finally, although statistically significant differences were identified, the absolute magnitude of these differences is relatively small (approximately 1 mm). Despite the high reproducibility demonstrated in our reliability analysis, these small variations require careful interpretation in terms of their clinical impact. Future studies incorporating prospective designs, larger cohorts, and intraoperative correlation are warranted to validate these findings and further clarify their surgical relevance.

Therefore, these findings highlight the presence of interindividual anatomical variability in the thyroid cartilage, which may be relevant for preoperative assessment in medialization thyroplasty. We can assume that the observations made on CT scans can be extrapolated to real-life patients, but we are currently working on comparing our results in CT scans to in vivo situations, with intraoperative measurements of the thyroid lamina.

In recent years, there has been increasing interest in treatment personalization tailored to individual patients, allowing for the development of structured preoperative planning strategies [18,19,20,21,22]. Advances in computed tomography imaging enable detailed visualization of patient-specific laryngeal anatomy, which may support the adaptation of existing implants or the design of customized laryngeal prostheses based on individual anatomical landmarks. In this setting, three-dimensional (3D) modeling and additive manufacturing technologies, including 3D printing, have been proposed as potential tools to translate radiological data into patient-specific implant designs. In this context, we are currently working on CT-based and 3D-assisted approaches that may contribute to improved implant fit and reduced intraoperative adjustments; however, further research is required to determine their reproducibility, clinical feasibility, and impact on functional outcomes. Ongoing studies continue to evaluate the role of these technologies within a precision medicine framework for laryngeal framework surgery.

## Data Availability

The original contributions presented in this study are included in the article/Supplementary Material. Further inquiries can be directed to the corresponding authors.

## Supplementary Materials

The following supporting information can be downloaded at: https://www.mdpi.com/article/10.3390/jpm16060294/s1, File S1: Detailed statistical analysis outputs, including Shapiro-Wilk normality tests, Wilcoxon signed-rank and Mann-Whitney U test results, full generalized linear mixed model output with Risk Ratios and likelihood ratio tests, post hoc power analysis, and software details.
