# Supplementary material for: Interindividual Variability in Thyroid Cartilage Lamina Width and Its Implications for Personalized Medialization Thyroplasty
Source: J Pers Med. 2026 May 29;16(6):294. doi: 10.3390/jpm16060294 (PMC13302219; doi:10.3390/jpm16060294)
Supplement: Supplementary file 1 [file jpm-16-00294-s001.zip › jpm-4149977-supplementary.pdf]

Supplementary Material

Interindividual Variability in Thyroid Cartilage Lamina Width and Its Implications for Personalized Medialization Thyroplasty

File S1: Detailed Statistical Analysis Outputs

S1. Normality Assessment (Shapiro-Wilk Test)

Prior to statistical analysis, normality was assessed for the three key variables using the Shapiro-Wilk test. All three variables showed significant departure from normality ($p < 0.001$), confirming the appropriateness of non-parametric methods throughout the analysis.

Variable	W statistic	p-value
Anterior thickness (GROSOR_ANT)	W = 0.8274	$p = 1.95 \times 10^{-6}$ *
Posterior thickness (GROSOR_POST)	W = 0.8537	$p = 1.02 \times 10^{-5}$ *
Thickness difference (Post − Ant)	W = 0.7039	$p = 3.91 \times 10^{-9}$ *

** p < 0.05: significant departure from normality.*

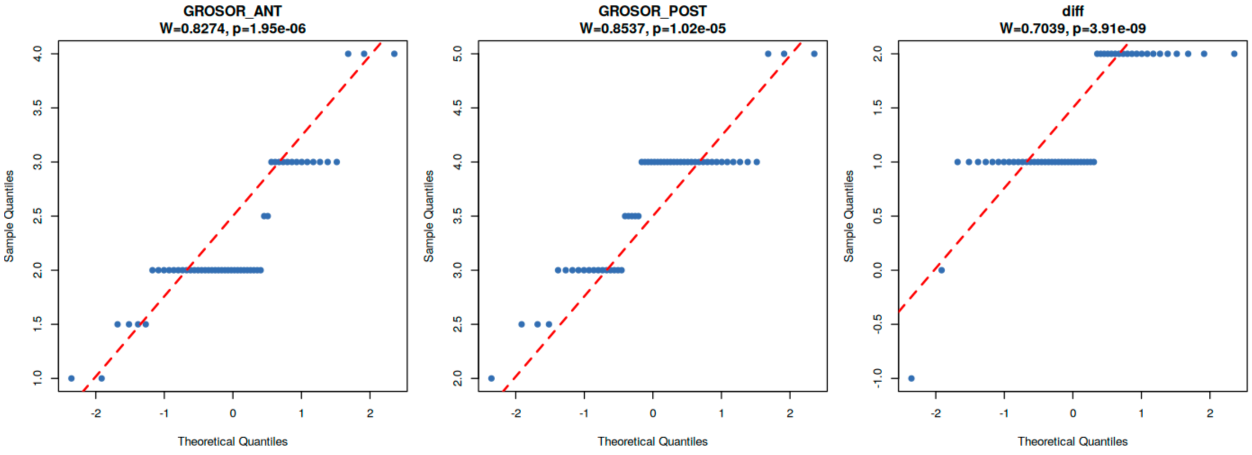

Figure S1. Q-Q plots for anterior thickness, posterior thickness, and their difference. Deviation from the reference line confirms non-normal distribution in all three variables.

S2. Non-Parametric Statistical Tests

S2.1 Wilcoxon Signed-Rank Test (Anterior vs. Posterior Thickness)

The Wilcoxon signed-rank test was used to compare anterior and posterior thyroid cartilage thickness within subjects, given the confirmed non-normal distribution.

Parameter	Value
Test statistic (V)	16.0
p-value	1.68×10^{-10}
Median anterior thickness	2.0 mm (IQR: 2.0–3.0)
Median posterior thickness	4.0 mm (IQR: 3.0–4.0)
Median difference (Post − Ant)	1.0 mm (IQR: 1.0–2.0)

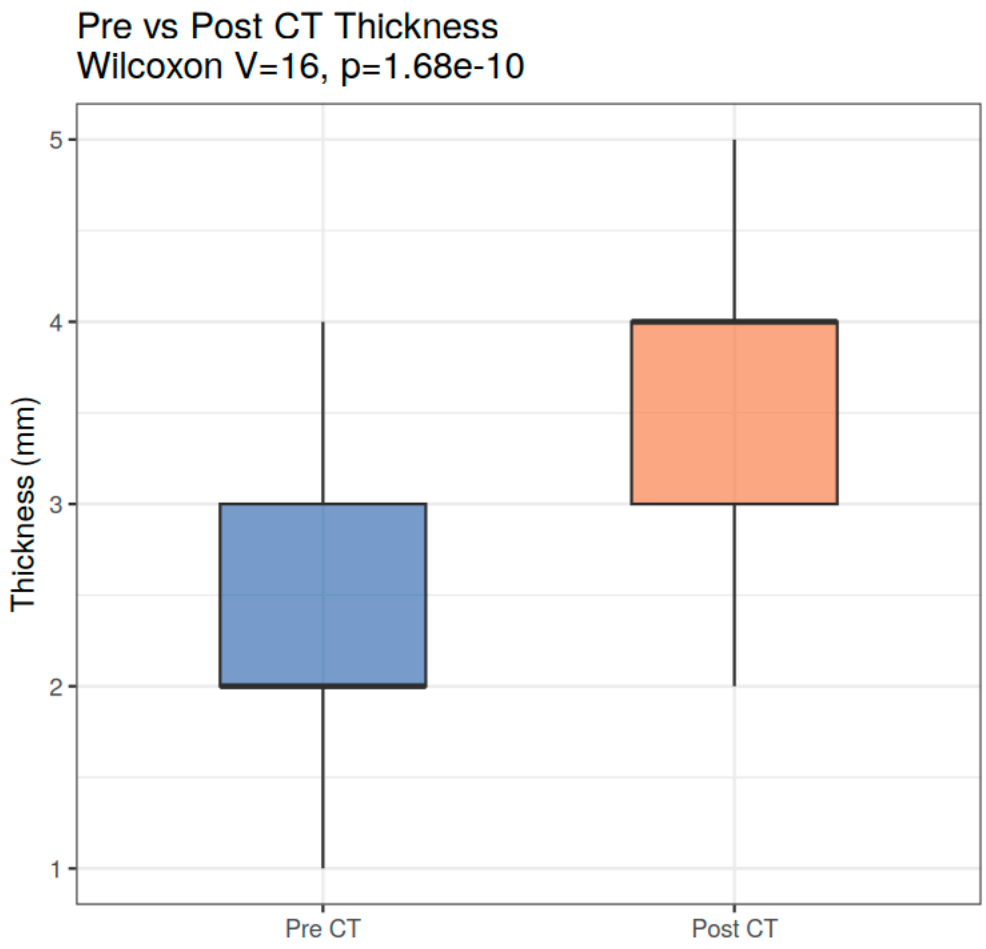

Figure S2. Boxplot comparing anterior (Pre-CT) and posterior (Post-CT) thyroid cartilage thickness. The posterior aspect is consistently greater than the anterior aspect (Wilcoxon V = 16, p = 1.68 × 10⁻¹⁰).

S2.2 Mann-Whitney U Test (Thickness Difference by Sex)

The Mann-Whitney U test was applied to assess whether the magnitude of the thickness difference (posterior − anterior) differed between female and male patients.

Parameter	Value
Test statistic (W)	387.5
p-value	0.404
Median difference — Female (n = 33)	1.0 mm (IQR: 1.0–2.0)
Median difference — Male (n = 21)	1.0 mm (IQR: 1.0–2.0)
Interpretation	No significant sex difference

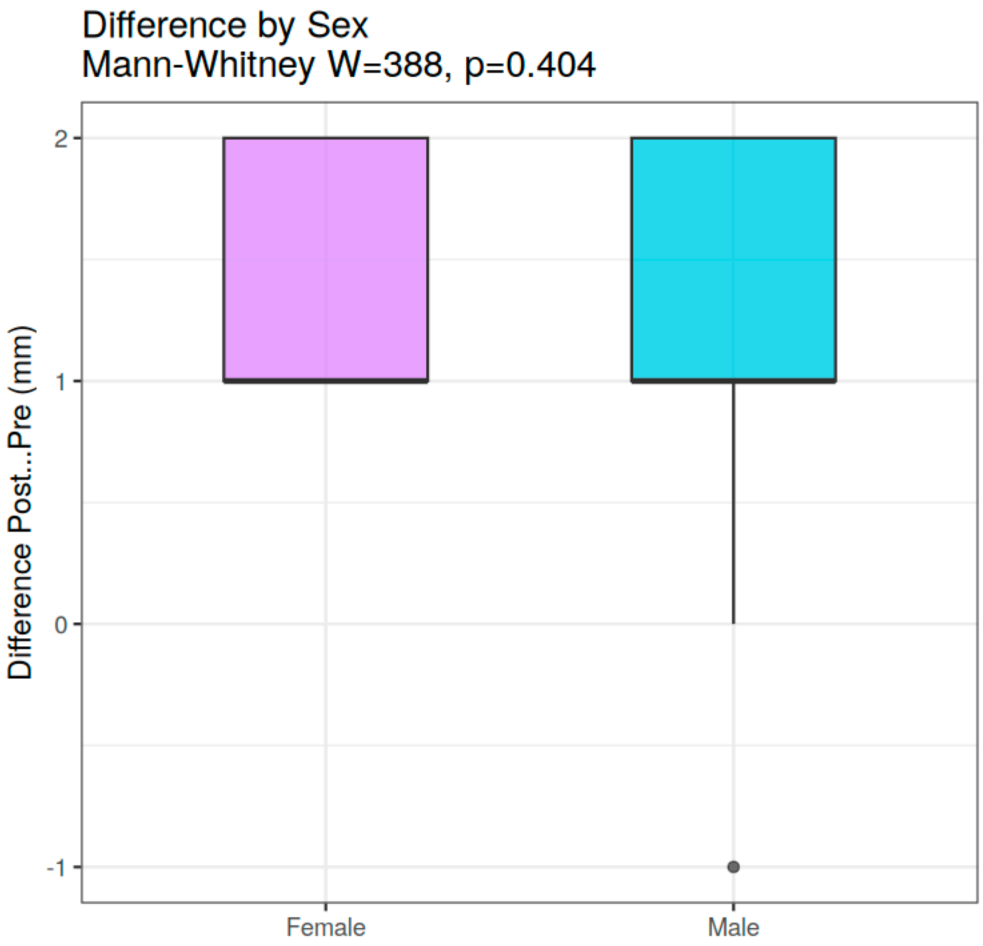

Figure S3. Boxplot of thickness difference (posterior − anterior) by sex. No significant difference was found between female and male patients (Mann-Whitney W = 387.5, p = 0.404).

S2.3 Spearman Rank Correlation (Thickness Difference vs. Age)

Spearman's rank correlation was used to evaluate the association between patient age and the magnitude of the anterior-posterior thickness difference.

Parameter	Value
Spearman rho	0.071
p-value	0.608
Interpretation	No significant correlation with age

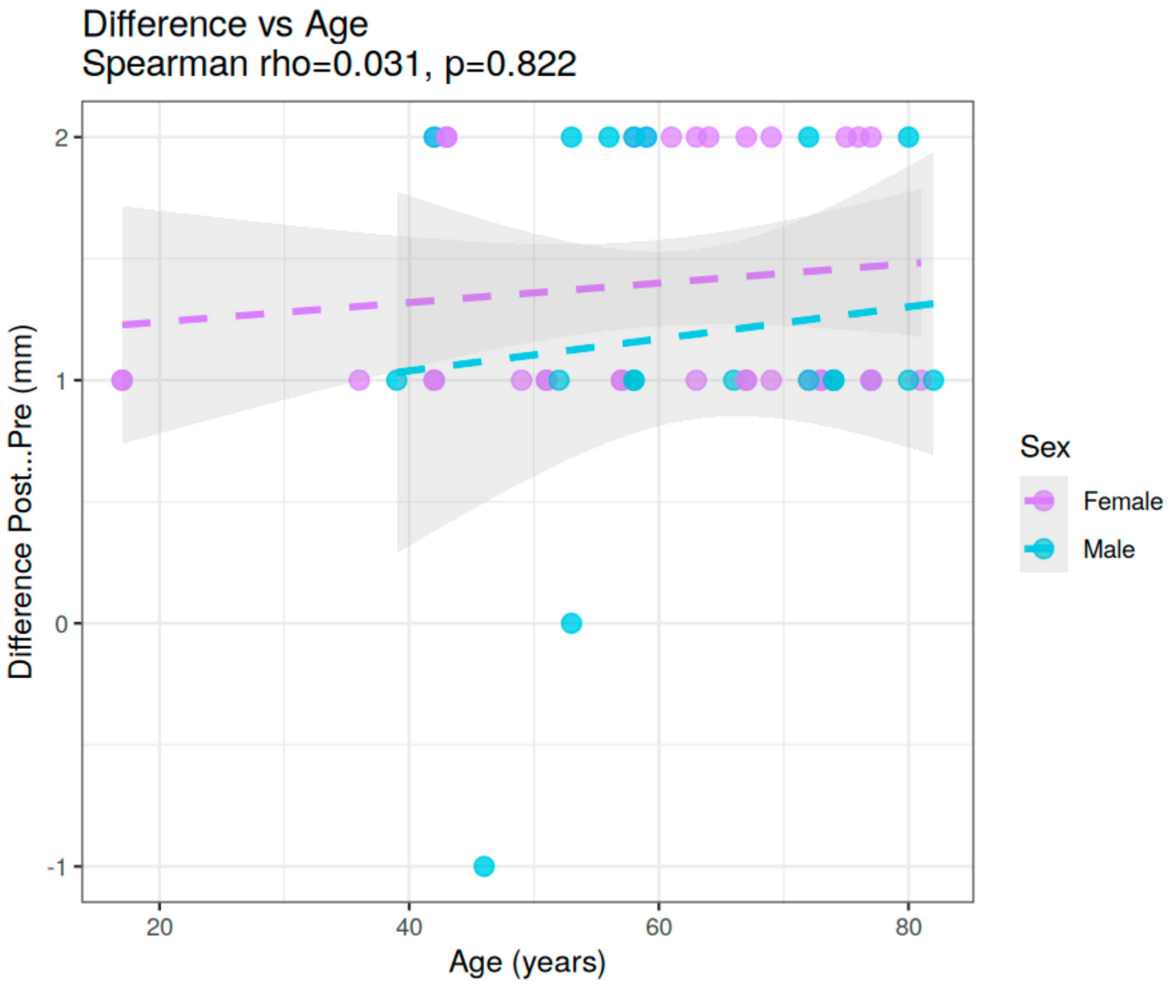

Figure S4. Scatter plot of thickness difference (posterior − anterior) versus age, by sex. Spearman's rho = 0.071 (p = 0.608), indicating no significant age-related trend.

S3. Generalized Linear Mixed Model (GLMM)

A generalized linear mixed model (GLMM) with Poisson family and log link was fitted to simultaneously evaluate the effects of anatomical region (anterior vs. posterior), age, and sex on cartilage thickness, accounting for the paired structure of the data by including patient identifier as a random effect.

S3.1 Full Model Output

Fixed Effect	Estimate	SE	z	p-value
(Intercept)	0.684	0.124	5.51	< 0.001 ***
REGION (Posterior)	0.535	0.156	3.43	0.001 ***
Age (centered)	0.007	0.006	1.04	0.296
Sex (Male)	0.275	0.184	1.50	0.135
REGION × Age	−0.001	0.008	−0.14	0.885
REGION × Sex	−0.161	0.236	−0.68	0.494

S3.2 Risk Ratios with 95% Confidence Intervals

Parameter	RR	95% CI (low)	95% CI (high)
(Intercept)	1.981	1.554	2.527
REGION (Posterior)	1.707	1.257	2.319
Age (centered)	1.007	0.994	1.019
Sex (Male)	1.317	0.918	1.889
REGION × Age	0.999	0.983	1.015
REGION × Sex	0.851	0.536	1.351

S3.3 Likelihood Ratio Tests

Model comparison was performed using likelihood ratio tests (LRT) to assess the contribution of interaction terms and main effects.

Comparison	Result
Full model vs. main effects only ($\chi^2(2)$)	0.526, $p = 0.769$ — interactions not significant
Main effects vs. null model ($\chi^2(3)$)	21.673, $p < 0.001$ — main effects significant

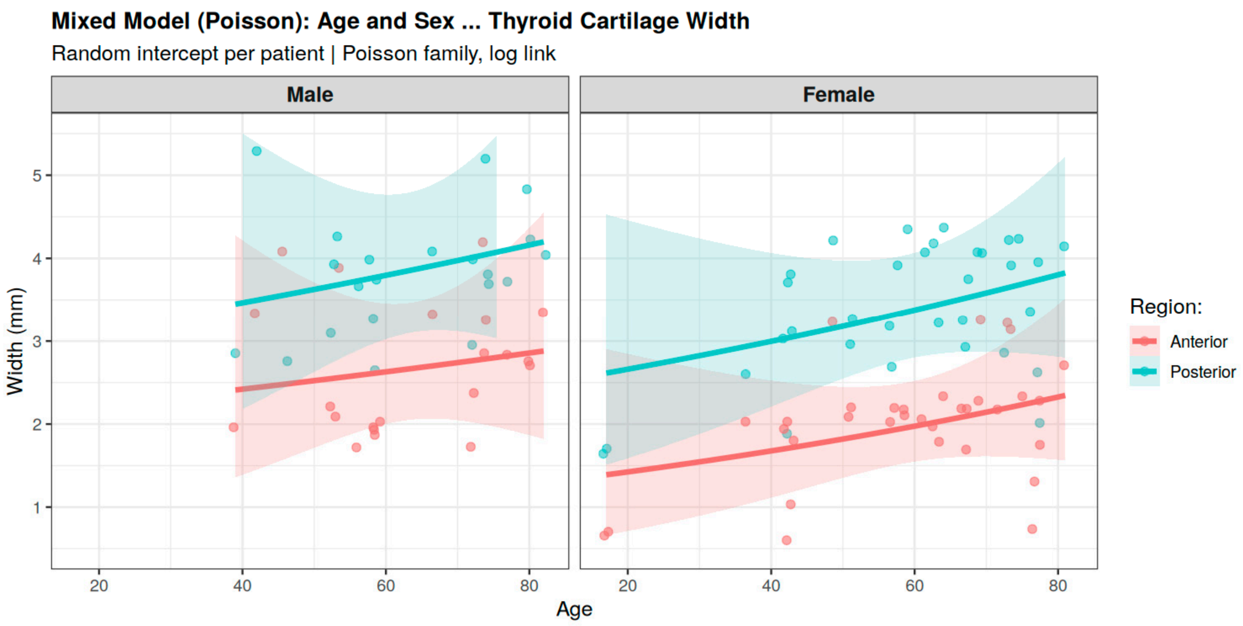

Figure S5. GLMM visualization: predicted cartilage width (mm) by age, anatomical region (anterior/posterior), and sex (female/male). Fitted curves represent Poisson GLM predictions with 95% confidence bands.

S4. Post Hoc Power Analysis

A post hoc power analysis was conducted based on the primary outcome (difference between anterior and posterior thyroid cartilage thickness). The analysis confirmed adequate statistical power (> 80%) to detect the observed effect size at a significance level of α = 0.05, supporting the validity of the primary findings despite the relatively modest sample size (n = 54 paired observations from 47 patients).

Parameter	Value
Sample size (paired observations)	54
Observed median difference	1.0 mm (IQR: 1.0–2.0)
Test used	Wilcoxon signed-rank
Significance level (α)	0.05
Estimated power	> 80%

S5. Software and Reproducibility

All statistical analyses were performed using R statistical software (version 4.3.1; R Core Team, 2023; R Foundation for Statistical Computing, Vienna, Austria) and RStudio (version 2024.12.1+563; Posit Software, PBC, Boston, MA, USA). The following R packages were used:

Package	Version	Purpose
lme4	≥ 1.1	Generalized linear mixed models (glmer)
ggplot2	≥ 3.4	Data visualization
dplyr	≥ 1.1	Data manipulation
tidyr	≥ 1.3	Data reshaping
readxl	≥ 1.4	Excel data import

The complete R script used for all analyses is available upon request from the corresponding author.
